# Prescription drug claims following a traumatic spinal cord injury for older adults: a retrospective population-based study in Ontario, Canada

**DOI:** 10.1038/s41393-018-0174-z

**Published:** 2018-07-31

**Authors:** Sara J. T. Guilcher, Mary-Ellen Hogan, Andrew Calzavara, Sander L. Hitzig, Tejal Patel, Tanya Packer, Aisha K. Lofters

**Affiliations:** 1 Leslie Dan Faculty of Pharmacy, University of Toronto, Toronto, ON Canada; 2 Institute for Clinical Evaluative Sciences, Toronto, ON Canada; 3 Centre for Urban Health Solutions, St. Michael’s Hospital, Toronto, ON Canada; 4 Rehabilitation Sciences Institute, Faculty of Medicine, University of Toronto, Toronto, ON Canada; 5 St. John’s Rehab Research Program, Sunnybrook Research Institute, Sunnybrook Health Sciences Centre, Toronto, ON Canada; 6 Neural Engineering and Therapeutics Team, Toronto Rehabilitation Institute, University Health Network, Toronto, ON Canada; 7 Department of Occupational Science and Occupational Therapy, Faculty of Medicine, University of Toronto, Toronto, ON Canada; 8 Michael G. DeGroote School of Medicine, McMaster University, Toronto, ON Canada; 9 Schlegel–University of Waterloo Research Institute of Aging, Toronto, ON Canada; 10 Waterloo Institute for Complexity and Innovation, Toronto, ON Canada; 11 University of Waterloo School of Pharmacy, Toronto, ON Canada; 12 School of Occupational Therapy, Dalhousie University, Halifax, NS Canada; 13 Department of Rehabilitation, Radboud University Medical Centre, Nijmegen, Netherlands; 14 School of Health Administration, Dalhousie University, Halifax, NS Canada; 15 Department of Family and Community Medicine, University of Toronto, Toronto, ON Canada

## Abstract

**Study design:**

Retrospective cohort study.

**Objectives:**

The objectives for this study were to examine the prevalence of polypharmacy for people with traumatic spinal cord injury (SCI) following injury and to determine risk factors.

**Setting:**

Ontario, Canada

**Methods:**

We used provincial-level administrative health services data of publicly funded healthcare encounters housed at the Institute for Clinical Evaluative Sciences, Toronto, Ontario. We examined prescription medications dispensed over a 1 year period post injury for persons 66+ years with an index traumatic SCI between 2004 and 2014. Polypharmacy was defined as being on 10 or more drug classes. Descriptive and analytical statistics were conducted. Relative risks and 95% confidence limits for factors related to polypharmacy were calculated using a robust Poisson multivariate regression model.

**Results:**

We identified 418 cases of persons with traumatic SCI during the observation window. A total of 233 patients (56%) were taking at least 10 drug classes in the year following discharge from care for traumatic SCI. The mean number of drug classes taken post injury was 11 (SD = 6). Continuity of care was significantly associated with polypharmacy, with a higher continuity of care (having at least 75% of visits with the same doctor) reducing the risk of polypharmacy. The most common drugs prescribed were laxatives, opioids and cardiovascular-related drugs.

**Conclusion:**

Findings suggest that polypharmacy is extensive among older adults with traumatic SCI. Persons with better continuity of care are less likely to have polypharmacy compared to those with less continuity.

**Sponsorship:**

This project was funded by a Connaught New Investigator Award (University of Toronto), and the Craig H. Neilsen Foundation Psychosocial Research Pilot Grant (Grant #441259).

## Introduction

A traumatic spinal cord injury (SCI) is a devastating injury that causes significant burden to individuals, their family members, and society [1, 2]. The age of incidence of SCI is bimodal, with peaks at the third and sixth decade [3]. The incidence of traumatic SCI among older adults is expected to rise with an aging population and is mainly due to unintentional falls [3, 4]. Irrespective of age, episodic secondary health complications are common among persons with SCI, such as spasticity, urinary tract infections, pressure sores, respiratory infections [1, 2]. Other chronic conditions often include overuse upper extremity injuries, bowel and bladder problems, sleep disorders, chronic pain, fatigue, depression, diabetes, heart disease, and osteoarthritis [2, 5].

Importantly, adults with the onset of a SCI at an older age may have worse functional outcomes compared to younger adults [6–8]. Moreover, mortality rates post SCI are higher among older adults, with a hazard ratio of 1.08 for each 1 year increase in age at the time of injury [8]. Reasons for increased mortality are not well understood, however pre-existing co-morbidities may account for worse functional outcomes and mortality rates [9]. Aging with a SCI has been associated with increased number of complications and conditions [2]; particularly with bladder and bowel dysfunction [7, 9]. These episodic complications and chronic conditions can have substantial impact on this population’s overall health and wellbeing, re-integration into the workplace, and quality of life [10, 11].

The management of these complications and chronic conditions often include pharmacotherapy [12]. The classes of medications typically prescribed for the most common complications and conditions include: (1) antispasmodics; (2) benzodiazepines and non-benzodiazepine hypnotics; (3) narcotic analgesics; (4) anticonvulsants; (5) selective serotonin reuptake inhibitors, serotonin-norepinephrine reuptake inhibitors and tricyclic antidepressants; and (6) skeletal muscle relaxants [12]. While minimal research exists on this topic for SCI, preliminary work [12–15] has suggested a relatively high prevalence of polypharmacy, often defined as the concurrent use of five or more medications [16]. Medication use following a SCI has been shown in a small prospective study (*n* = 72 patients) to increase approximately three times the number of medications used prior to injury [15]. Recently, Kitzman and colleagues published a population-based study using administrative health data in the United States examining the prevalence of polypharmacy, and drug-related problems over a 3 year period, which included adverse drug events and medication errors secondary to drug-drug, drug-disease, or drug-nutrient reactions [12]. Findings showed significant polypharmacy (56%) among persons with a SCI. Exploratory in nature, the analysis was not stratified by sex, and did not examine the factors potentially driving polypharmacy and drug-related problems. Recently, Hand and colleagues examined polypharmacy and adverse drug events among persons with SCI in the United States using administrative data with private drug insurance claims [14]. Compared to propensity-score matched controls, Hand et al demonstrated that persons with SCI are at increased risk for adverse drug related events, particularly women, those with comorbidities and those on multiple medications [14].

A recent chart review of a primary care clinic in Ontario (Canada), which specializes in services for persons with mobility issues, identified substantial medication-related problems for persons with SCI (*n* = 19) [13]. The most common medication-related problems were untreated pain (23%), ineffective medications (21%), adverse drug reactions (18%), and under or over-dosage conditions (9%), respectively. Polypharmacy, including prescription medications and over-the-counter medications, was identified in 63% of the patient charts reviewed; increasing to 74% when natural health products were included. Additionally, this pilot study identified that at least one high-risk medication (e.g., muscle relaxants, anticonvulsants, antidepressants, and benzodiazepines) was prescribed for 15 of the 19 patients (79%).

To date, there is limited research, particularly in Canada, examining the extent to which polypharmacy and drug-related problems influence medication adherence, health and well-being outcomes, health care utilization, and direct patient and health system costs. Among the general population, the complexity of pharmacotherapy renders persons at risk for drug-related problems, potentially impacting morbidity and decreasing life expectancy [17]. When a SCI is factored into the equation, there are likely additional negative consequences. For instance, persons with SCI may have increased risk of drug-related problems due to evidence of altered drug absorption, distribution, metabolism and elimination, and few pharmacokinetic studies have been conducted in persons with SCI [18]. Furthermore, persons with SCI are at risk of pharmacodynamic drug interactions because they may take several drugs with overlapping mechanisms of action. This may result in a drug’s effect being blocked or enhanced, resulting in lack of efficacy or increased adverse effects.

Overall, polypharmacy is under-examined but of significant concern post-SCI, which requires more research on the prevalence, cause and related outcomes to inform best practices for this population. Previous research has shown poor functional outcomes and high mortality rates following older onset SCI; however, reasons for these differences are poorly understood. To address this gap of research on older adults with SCI, this study examined the prevalence of polypharmacy post-SCI for older adults and the factors associated with polypharmacy using population-based administrative health data in Ontario, Canada.

## Methods

We used a retrospective cohort design, drawing from administrative health data housed at the Institute for Clinical Evaluative Sciences (ICES), Toronto, Ontario. The datasets contain routinely collected records of all publicly funded health care encounters within the province of Ontario, Canada. Ontario has over 13 million residents, representing approximately 40% of the Canadian population, and has a universal health system that funds all medically necessary care by physicians, hospitals, inpatient rehabilitation and some other care. A comprehensive list of drugs (www.formulary.health.gov.on.ca) is publicly funded for those 65 years and older. Those under 65 years who receive social assistance or have catastrophic drug costs are also funded on the public drug program. We limited this analysis of polypharmacy to those over 66 years because of access to complete drug records.

Hospitalization records (Discharge Abstract Database, DAD) provided admission and discharge dates, transfer information, and the most responsible diagnosis and up to 24 secondary diagnostic codes (based on International Classification of Disease, Tenth Revision Canada, ICD-10-CA codes). The National Rehabilitation Reporting System (NRS) provided admission and discharge dates, transfer information, and diagnostic codes for those who received in-patient rehabilitation care. The National Ambulatory Care Reporting System (NACRS) provided diagnostic codes for all visits to the emergency department, same day surgeries, and high volume ambulatory care clinic visits. Outpatient physician visit information was obtained from the Ontario Health Insurance Plan (OHIP) database. Physician specialty, service date and location, and diagnostic codes were obtained from this database. The Ontario Drug Benefits database (ODB) provided records of drugs dispensed outside of hospitals and rehabilitation facilities. Each drug product in Canada has a unique identifier (drug identification number, DIN) and drugs of the same class (e.g., HMG-CoA reductase inhibitors) were grouped together when determining the number of drugs an individual received. The Ontario Registered Persons database provided basic demographic information (e.g., sex, age, date of birth, residential postal code), and vital statistics information, including death date, for each Ontario resident. More information on the datasets can be found at datadictionary.ices.on.ca. Ontario’s health administrative data has demonstrated validity and reliability. Individuals within the datasets were linked using unique encoded identifiers and analyzed at ICES.

### Research ethics

The use of data in this project was authorized under section 45 of Ontario’s *Personal Health Information Protection Act*, which does not require review by a Research Ethics Board. However, this study was approved by the University of Toronto Research Ethics Board.

### Study population

**Fig. 1:**
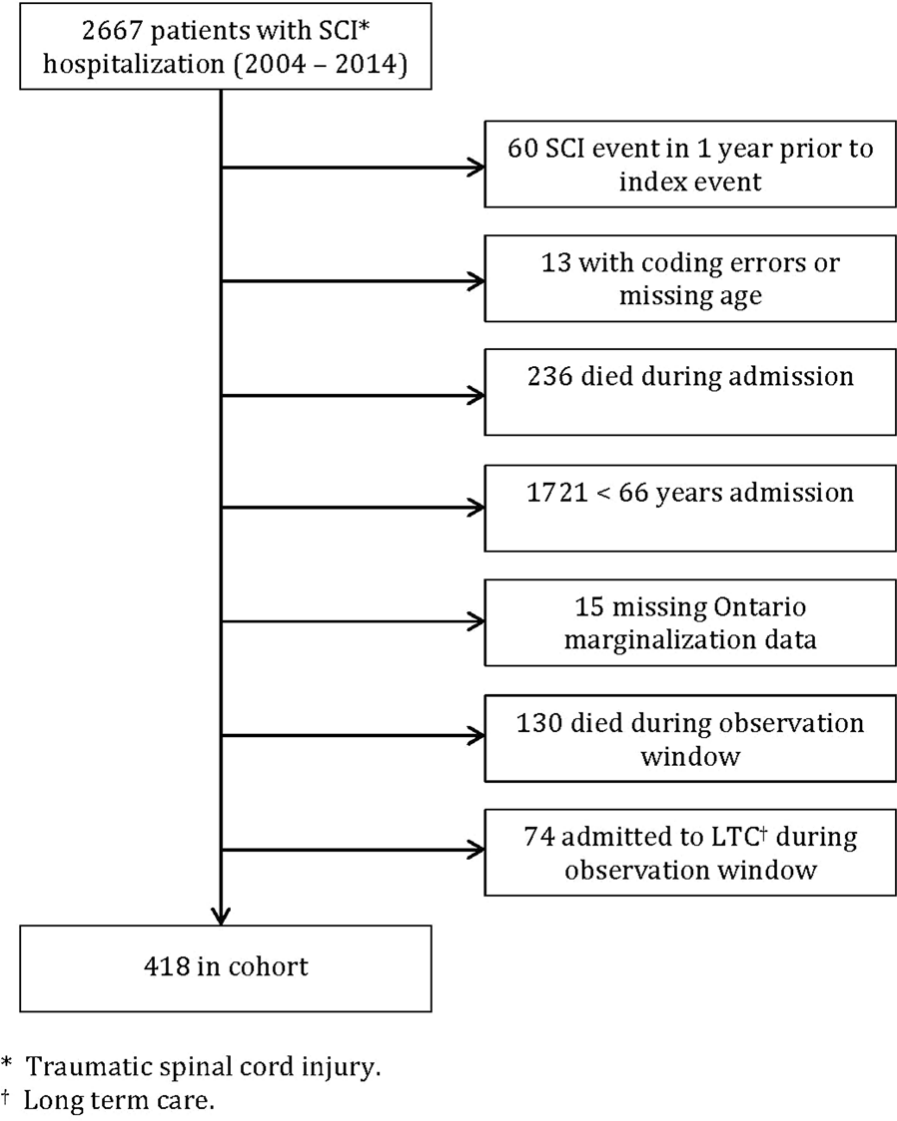
Participant flow chart. Prior to exclusions, we identified 2667 persons with traumatic spinal cord injury (SCI) between 2004 and 2014. After exclusions, we had a final cohort of 418 individuals ≥66 years of age with complete data and alive during our observation window

Individuals who were hospitalized with their initial SCI between April 1, 2004 and March 31, 2014 and were at least 66 years of age at the time of hospitalization injury were included. We used a 66 year cut-off to ensure we had a full year pre-injury to assess medication use (see Supplemental file, Appendix for ICD-10 codes). Individuals were excluded if there was a SCI event in the year prior to admission, if they died during their initial admission, if they died or were admitted to long-term care during the 1 year following hospital discharge, if coding errors were present or if key demographic information was missing (see Fig. 1 for the complete list). We used an incident cohort of persons with traumatic SCI to ensure they were comparable and at a similar stage in their course of injury.

### Study variables

#### Main outcome of interest: polypharmacy

Our primary outcome was the prevalence of polypharmacy within the SCI population (at least 66 years of age) during the 1 year after discharge home from the acute hospital or a rehabilitation institution. Although it is common to use 5 or more drugs as a threshold for polypharmacy among older adults, most individuals with SCI received at least 5 drugs during the observation period. Therefore, we used a threshold of 10 or more drug classes to examine factors related to receipt of higher numbers of prescription drugs. We used cumulative polypharmacy, which is an established approach for large administrative databases [19]. All unique drug classes received were summed over the 1-year observation periods (pre and post index injury). If the same drug class was received more than once during the observation period, it was counted only once. Drug classes were defined as drugs with similar therapeutic or pharmacologic use, for example, beta blockers, diuretics and opioids are distinct classes, and were based on an adaptation of the American Hospital Formulary Service classification system.

#### Independent variables of interest

Our main independent variables of interest were sex, age, multimorbidity, continuity of care and material deprivation. We also included the following variables in the models to control for potential confounding: length of stay in inpatient rehabilitation, level of injury and number of drug classes used in the year before the index SCI. The Johns Hopkins ACG® System Version 10 was used to categorize individuals by morbidity burden. This proprietary software determined the number of ACG® System Aggregated Diagnosis Groups (ADGs) using hospitalization, emergency department and physician office visits for the 2 year period prior to SCI. Higher numbers of ADGs indicated greater morbidity. Continuity of care is a concept that assumes a patient sees a physician or other care provider multiple times over a period of time; repeated encounters allow the physician to develop and carry out a care plan. Continuity of care was calculated for anyone with at least 3 ambulatory physician visits. The proportion of primary care physician visits (office, home, phone) made to the primary care provider seen most often in the year following the index injury was determined, where a threshold of 75% of visits with the same physician was used to indicate high continuity and less than 50% of visits made to the same physician was defined as low continuity [20]. Material deprivation is a dimension of the Ontario Marginalization Index. It uses census information to calculate scores for geographical areas that reflect education attainment, single parent families, government transfer payments, unemployment, income, and homes in poor repair [21]. We determined the neighborhood income quintile for each subject using their postal code and census data but did not include it in the regression models because of high multicollinearity with material deprivation. We also calculated the proportion of individuals with the following chronic diseases to provide descriptive information about the cohort: asthma, congestive heart failure (CHF), chronic obstructive pulmonary disease (COPD), hypertension, diabetes, rheumatoid arthritis and dementia using established diagnostic codes and drugs.

### Statistical analysis

Variables were described using means, standard deviations and proportions. Chi squared and *t*-tests were used to test for differences between groups. The Cochran-Armitage test was used to test differences between groups for ordinal data. Wilcoxon sign rank tests were used to test for differences in polypharmacy and continuity of care before and after injury. McNemar tests were used to calculate differences in chronic disease before and after injury. An alpha of less than 0.05 was considered statistically significant. Relative risks and 95% confidence limits for factors related to polypharmacy were calculated using a robust Poisson multivariate generalized estimating equations regression model [22]. As a sensitivity analysis, the regression analysis was repeated using those excluded due to death or transfered to long-term care. Persons who died were excluded to ensure complete medication use during the observation window. Individuals in long-term care were excluded due to significant differences in patient characteristics and care provision. All data were analyzed with SAS version 9.4 (SAS Institute Inc., Cary, NC; www.sas.com).

## Results

After applying inclusion and exclusion criteria, there were 418 individuals 66 years of age and older in Ontario who had an initial traumatic SCI between April 1, 2004 and March 31, 2014 (Fig. 1). The mean age (SD) at injury was 75 (6) and 63% were male. Women were older than men on average [76 (6) vs. 74 (6), *p* < 0.01]. The level of injury was cervical in 77% of patients. The primary cause of injury was a fall (74% of patients), followed by a motor vehicle accident (16% of patients). Patients spent a median (IQR) of 19 (11–36) days in an acute hospital after their initial injury and 58% were transferred to inpatient rehabilitation. More men than women were transferred to inpatient rehabilitation (62 vs. 51%, *p* < 0.05). Overall median (IQR) length of stay in a rehabilitation facility for those who went to rehabilitation (*n* = 241) was 66 (40–99) days. The mean (SD) number of drug classes taken in the year before SCI was 7.7 (5.7) and was significantly larger for women compared to men [8.8 (6.3) vs. 7.1 (5.2), *p* < 0.01]. Similarly, the mean number of pre-injury ADGs was larger for women compared to men [10.2 (4.1) vs. 8.9 (4.0), *p* < 0.01]. Additional details can be found in Table 1.

**Table 1:** Population characteristics of individuals ≥ 66 years of age with a traumatic spinal cord injury (fiscal years 2004–2014), Ontario, Canada (*n* = 418)

	Overall *n* = 418	Polypharmacy (≥ 10 drug classes) *n* = 233	< 10 drug classes *n* = 185	Females *n* = 153	Males *n* = 265
Female, *n* (%)	153 (37)	88 (38)	65 (35)	153 (100)	NA
Age at injury, mean (SD)	75 (6)	74 (6)	75 (7)	76 (6)	74 (6)**
Injury level, *n* (%)					
1 (cervical)	321 (77)	173 (74)	148 (80)	110 (72)	211 (80)
2 to 4 (thoracic, lumbar, sacral)	97 (23)	60 (26)	37 (20)	43 (28)	54 (20)
Income quintile, *n* (%)					
1 (low)	72 (17)	32 (17)	40 (17)	29 (19)	43 (16)
2	94 (22)	45 (24)	49 (21)	37 (24)	57 (22)
3	81 (19)	31 (17)	50 (22)	26 (17)	55 (21)
4	77 (18)	34 (18)	43 (18)	29 (19)	48 (18)
5 (high)	94 (22)	43 (23)	51 (22)	32 (21)	62 (23)
Deprivation quintile, *n* (%)^a^					
1 (least deprived)	93 (22)	49 (21)	44 (24)	36 (24)	57 (22)^*^
2	99 (24)	54 (23)	45 (24)	29 (19)	70 (26)
3	82 (20)	48 (21)	34 (18)	23 (15)	59 (22)
4	85 (20)	52 (22)	33 (18)	41 (27)	44 (17)
5 (most deprived)	59 (14)	30 (13)	29 (16)	24 (16)	35 (13)
Continuity of care with a single health care provider, *n* (%)^b^					
<50% (low continuity of care)	251 (60)	154 (66)	97 (52)**	53 (35)	99 (37)
50–75%	126 (30)	65 (28)	61 (33)	62 (41)	108 (41)
>75% (best continuity of care)	41 (10)	14 (6)	27 (15)	38 (25)	58 (22)
ADG quintile, *n* (%)^c^					
1 (low comorbidity)	70 (17)	28 (12)	42 (23)**	18 (12)	52 (20)**
2	119 (28)	58 (25)	61 (33)	33 (22)	86 (32)
3	63 (15)	42 (18)	21 (11)	26 (17)	37 (14)
4	94 (22)	55 (24)	39 (21)	39 (25)	55 (21)
5 (high comorbidity)	72 (17)	50 (21)	22 (12)	37 (24)	35 (13)
Pre-injury drug classes, mean (SD)	8 (6)	10 (6)	5 (4)**	10 (7)	8 (6)**
In-patient rehab, *n* (%)	241 (58)	146 (63)	95 (51)*	78 (51)	163 (62)*
Days in rehab, median (IQR)^d^	66 (40–99)	76 (42–107)	56 (35–86)**	59 (30–81)	72 (42–107)**

*NA*  not applicable

^a^Material deprivation is a dimension of the Ontario Marginalization Index and incorporates education attainment, single parent families, government transfer payments, unemployment, income, and homes in poor repair.

^b^ $${\mathrm{Continuity}\,{of}\,{care = }}\frac{{{\mathrm{all}\,{physician}\,{office}\,{visits}\,{to}\,{usual}\,{source}\,{of}\,{care }}\,\left( {n} \right)}}{{{\mathrm{all}\,{physician}\,{office}\,{visits}}\,\left( {n} \right)}}$$

^c^ADG = Aggregated diagnosis groups, Johns Hopkins ACG® system. A system to classify health conditions in administrative health data over 2 years pre-injury. Larger numbers of ADGs reflect more comorbidity.

^d^For those who were admitted to inpatient rehabilitation (*n* = 241)

^*^p  < 0.05

^**^p < 0.01

### Post-injury polypharmacy

A total of 364 (87%) patients were taking at least 5 drug classes and 233 patients (56%) were taking at least 10 drug classes in the year following discharge from care for SCI. The mean number of drug classes taken was 11 (6). When comparing those with post-injury polypharmacy (≥10 drug classes) to those with fewer post-injury drugs (<10 drugs classes), no statistical differences were seen for age, sex, level of injury, income or material deprivation. Significantly more patients with post-injury polypharmacy were admitted to inpatient rehabilitation (63 vs. 51%, *p* < 0.05) than those without polypharmacy and their median length of stay was longer [76 (42–107) days vs. 56 (35–86) days, *p* < 0.01]. More patients in the polypharmacy group had low continuity of care after SCI [66 vs. 52%, *p* < 0.01 (Table 1)].

### Pre-injury compared to post-injury

There was a significant increase in the number of drug classes taken post-injury vs. pre-injury (*p* < 0.01). Individuals with post-injury polypharmacy were taking a larger number of drugs before their injury than those without polypharmacy (mean pre-injury drug classes 9.9 (5.7) vs. 4.9 (4.2), *p* < 0.01). Similarly, the mean number of pre-injury ADGs was larger in those with post-injury polypharmacy (8.5 (4.0) vs. 10.1 (4.0), *p* < 0.01). Among the cohort, four of seven chronic conditions (congestive heart failure, chronic obstructive pulmonary disease, hypertension, diabetes) had greater prevalence post-injury vs. pre-injury. Continuity of care decreased significantly among patients post-injury compared to pre-injury for the overall cohort. See Table 2 for additional details.

**Table 2:** Prescribed medications, chronic conditions and continuity of care pre- and post-injury, for individuals ≥ 66 years of age with a traumatic spinal cord injury (fiscal years 2004–2014), Ontario, Canada (*n* = 418)

	≥ 66 years of age (*n* = 418)		*p*
	1 year pre-injury	1 year post-injury	
Prescribed drug classes, *n* (%)^a^			
0	28 (7)	23 (6)	<0.01
1–4	110 (26)	31 (7)	
5–9	137 (33)	131 (31)	
10–19	126 (30)	205 (49)	
20+	17 (4)	28 (7)	
Chronic conditions, *n* (%)^b^			
Asthma	53 (13)	57 (14)	0.125
Congestive heart failure	48 (11)	64 (15)	<0.01
Chronic obstructive pulmonary disease	45 (11)	63 (15)	<0.01
Hypertension	303 (72)	319 (76)	<0.01
Diabetes	135 (32)	144 (34)	<0.01
Rheumatoid arthritis	15 (4)	18 (4)	0.250
Dementia	26 (6)	26 (6)	1.00
Continuity of care, *n* (%)^a^			
<50%	152 (36)	251 (60)	<0.01
50–75%	170 (41)	126 (30)	
>75%	96 (23)	41 (10)	

^a^Wilcoxon sign rank test

^b^McNemar test

### Types of prescribed drugs post-injury

**Fig. 2:**
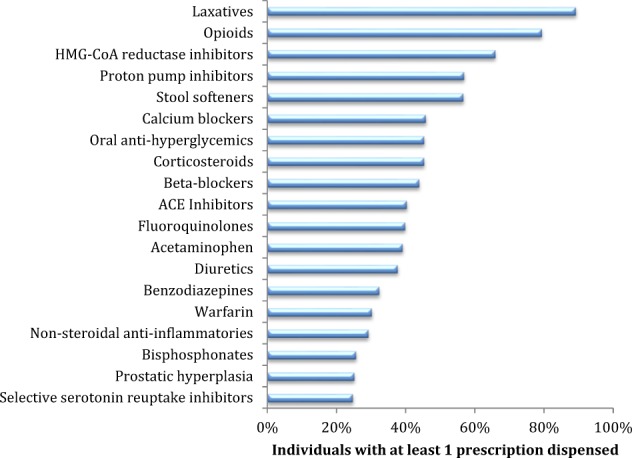
Most common drug classes used by individuals ≥66 years of age with a traumatic spinal cord injury (fiscal years 2004–2014), Ontario, Canada (*n* = 418). Only drug classes >25% are shown

The most common drug class prescribed post-injury was laxatives, with 89% of individuals having at least one prescription in the year following discharge. Opioids were the next most common, with 79% receiving at least one prescription. Drugs received by at least 25% of the SCI population are shown in Fig. 2. Eleven percent of the SCI population took drugs for overactive bladder. Seven of the top 40 drug classes were antibiotics.

### Regression analysis

**Fig. 3:**
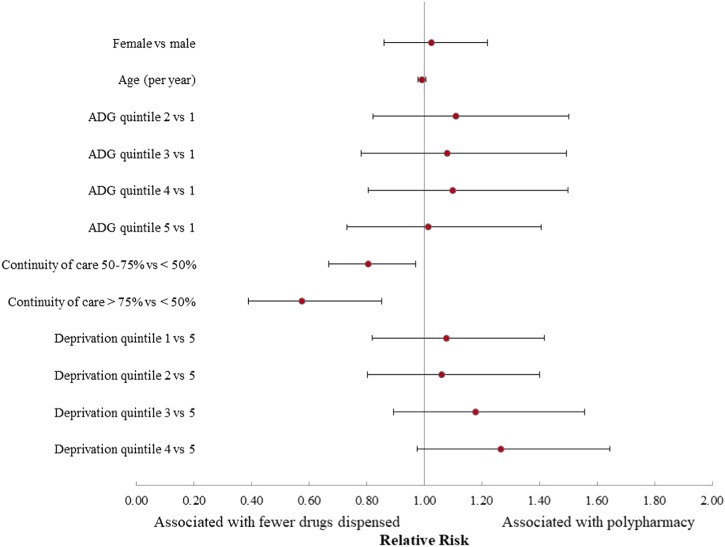
Risk of polypharmacy (10+ drugs) for individuals ≥66 years of age with a traumatic spinal cord injury (fiscal years 2004–2014), Ontario, Canada (*n* = 418). Relative risks greater than 1 are associated with polypharmacy. ADG quintile 1 = low comorbidity. Deprivation quintile 5 = most deprived. Results are adjusted for length of stay in rehabilitation facility, level of injury and number of pre-injury drugs

The association of independent variables on relative risk (RR) of polypharmacy (10+ drugs) post injury is shown in Fig. 3. Higher continuity of care (>75%) was associated with lower risk for polypharmacy compared to lower continuity (<50%) holding all other variables constant (RR = 0.58; 95% CI 0.39 to 0.85). Most of the independent variables included in the model such as sex, age, comorbidity and material deprivation were not significantly associated with risk of polypharmacy. When the sensitivity analysis was conducted including an additional 204 individuals who died or were admitted to long-term care, the same overall results were seen, with the exception of the continuity of care variable of 50–75 vs. <50% which was no longer significant in the sensitivity analysis.

## Discussion

Persons over age 66 with SCI are at risk of polypharmacy due the prevalence of secondary complications and co-occurrence of multimorbidity. Importantly, polypharmacy can increase the risk for drug-related problems, potentially impacting morbidity and mortality [12]. Despite these potential risks, there is limited research to date that has comprehensively examined pharmacoepidemiology for this population. In using administrative health data, our research contributes to the small body of work on this topic to date. Our findings show that there is substantial polypharmacy and multimorbidity among older adults with traumatic SCI, reinforcing the need for more research in this area.

Due to the majority of our cohort (87%) being prescribed 5+ drugs within the year post-injury, we increased the definition of polypharmacy to 10+ different drug classes. The overall mean number of drugs prescribed post-injury was 11. Our findings suggest older persons with SCI have higher prevalence of polypharmacy compared to the general older adult population [23]. According to the Canadian Institute for Health Information based on national data from 2016, 65.7% of older Canadians greater than 65 years of age were on 5+ drug classes, and 26.5% were on 10+ drug classes [23]. Comparatively, approximately twice as many persons in our SCI cohort were on 10+ drugs (56%) post-injury.

Our study found more health conditions such as congestive heart failure and chronic obstructive pulmonary disease post-injury compared to pre-injury. This may be a true effect, but may also be a result of increased healthcare contact following SCI and perhaps aging. This requires further investigation.

Interestingly, the only factor that significantly predicted polypharmacy in our model post-SCI was continuity of care. Previous research has shown that persons are at risk of polypharmacy with more prescribers involved in clinical care [24]. Moreover, better continuity of care is associated with high quality primary care [25–27]. Given the evidence supporting the value of care integration and coordination, there has been an emerging shift across health jurisdictions from solo practiced care to integrated medical homes. In this model, services are usually co-located or care is provided within different sites that have formalized integration. For example, in the United States the Patient-Centered Medical Homes are emerging as delivery models with the aim of improving continuity of care [28]. Persons with SCI would likely benefit from a similar model of care that involves colocation of services [29, 30], particularly with access to pharmacists who can provide comprehensive medication management [31].

Importantly, other sociodemographic factors such as material deprivation and sex were not significant predictors of polypharmacy. However, similar results have been reported for other populations whereby the effects of social determinants diminished for persons who are older and on the publicly funded drug plans [32]. Persons who are younger than 65 and who do not meet eligibility criteria for the public drug plans, need to pay out of pocket or rely on private insurance. While not specific to SCI, previous qualitative research has shown that access to medications for neurological populations is challenging for those who are not covered on the public drug plans [33].

Our results showed the top five most common drugs to be prescribed following an injury were laxatives, opioids, HMG-CoA reductase inhibitors (e.g., statins), proton-pump inhibitors (PPIs), and stool softeners. These findings are similar to previous published work [12, 13]. In chart reviews, Patel and colleagues found the most common reasons for medications were due to pain, constipation, muscle spasms, hypertension and depression [13]. Similarly, Kitzman and colleagues, using administrative data in the United States, identified higher prevalence of narcotics, anticonvulsants, and serontinergics compared to the control group [12]. One difference is that our study identified more cardiovascular drugs and PPIs, which may reflect the older age of our cohort among whom these drugs are common [23].

### Strengths and limitations

There are a few limitations to using administrative health data that should be noted. Firstly, we were not able to capture individuals who are on private drug coverage, those who pay out-of-pocket, prescriptions that were written but never dispensed, drugs dispensed in the hospital, over the counter medications and specific diagnoses or conditions for which prescriptions were written. Moreover, our methods cannot determine whether or not persons with SCI took the medications as prescribed. However, we estimate the majority of persons with SCI who are greater than 66 years old will be captured in the ODB database. Secondly, we only examined polypharmacy over a small window of time post injury. Thirdly, there are some drugs that were not part of the drug coverage program until later and present usage estimates may be higher. In addition, we cannot draw any conclusions about appropriateness of therapy or absent preventative therapies.

Despite these limitations, this study has several strengths. Using linked population administrative health data allowed us to capture data longitudinally for a population with relatively smaller prevalence, such as SCI. We also minimize attrition issues, with minimal loss to follow up. Most of the research to date on polypharmacy and SCI using administrative data has come from the United States with private drug coverage of relatively younger persons with SCI (18 to 64 years). This study provides additional evidence of polypharmacy of older adults with traumatic SCI with public drug coverage in Canada.

### Future directions

Future work would be warranted examining prescribed drugs over a longer follow-up window. Our team plans on conducting future research examining differences in polypharmacy post injury by type of injury (trauma vs. non- trauma), and classes of medications, duration of medications over time, and adverse drug-related events over a longer follow-up period. In addition, understanding the experiences with medications, effective strategies for optimal medication management would be beneficial to inform best practices, clinical management and patient experiences. Moreover, future work might examine the impact of polypharmacy on quality of life and overall well-being (e.g., patient reported outcomes).

### Summary

Findings of this study suggest that polypharmacy is extensive among older adults with traumatic SCI. Eighty-seven percent of patients took at least 5 drug classes and 56% took at least 10 drug classes. Persons with better continuity of care are less likely to have polypharmacy compared to those with less continuity. The most common drugs prescribed were laxatives, opioids and cardiovascular-related drugs.

## Electronic supplementary material

Appendix
