# Supplementary material for: Prescription drug claims following a traumatic spinal cord injury for older adults: a retrospective population-based study in Ontario, Canada
Source: Spinal Cord. 2018 Jul 31;56(11):1059–68. doi: 10.1038/s41393-018-0174-z (PMC6218396; doi:10.1038/s41393-018-0174-z)
Supplement: Supplementary file 1 — Appendix [file 41393_2018_174_MOESM1_ESM.docx]

**APPENDIX**

International Classification of Disease, 10^th^ Revision Canada (ICD-10-CA) codes for traumatic spinal cord injury cohort identification

| **Traumatic SCI Diagnosis Codes** |
| --- |
|  |
|  |
| *Cervical level* |
| S140 - Concussion and oedema of cervical spinal cord |
| S1410 - Complete lesion of cervical spinal cord |
| S1411 - Central cord lesion of cervical spinal cord |
| S1412 - Anterior cord syndrome of cervical spinal cord |
| S1413 - Posterior cord syndrome of cervical spinal cord |
| S1418 - Other injuries of cervical spinal cord |
| S1419 - Unspecified lesion of cervical spinal cord |
|  |
| *Thoracic level* |
| S240 - Concussion and oedema of thoracic spinal cord |
| S2410 - Complete lesion of thoracic spinal cord |
| S2411 - Central cord lesion of thoracic spinal cord |
| S2412 - Anterior cord syndrome of thoracic spinal cord |
| S2413 - Posterior cord syndrome of thoracic spinal cord |
| S2418 - Other injuries of thoracic spinal cord |
| S2419 - Unspecified lesion of thoracic spinal cord |
|  |
| *Lumbar Injury level* |
| S340 - Concussion and oedema of lumbar spinal cord |
| S3410 - Complete lesion of lumbar spinal cord |
| S3411 - Central cord lesion of lumbar spinal cord |
| S3412 - Anterior cord syndrome of lumbar spinal cord |
| S3418 - Other injuries of lumbar spinal cord |
| S3419 - Unspecified lesion of lumbar spinal cord |
|  |
| *Other* |
| S3430 - Laceration of cauda equina |
| S3438 - Other and unspecified injury of cauda equina |
| T060 - Injuries of brain and cranial nerves with injuries of nerves and spinal cord at neck level |
| T061 - Injuries of nerves and spinal cord involving other multiple body regions |
